# SleepOMICS: How Big Data Can Revolutionize Sleep Science

**DOI:** 10.3390/ijerph16020291

**Published:** 2019-01-21

**Authors:** Nicola Luigi Bragazzi, Ottavia Guglielmi, Sergio Garbarino

**Affiliations:** 1 Department of Health Sciences (DISSAL), Postgraduate School of Public Health, Genoa University, 16132 Genoa, Italy; 2 Department of Neuroscience, Rehabilitation, Ophthalmology, Genetics, Maternal and Child Health (DINOGMI), Genoa University, 16132 Genoa, Italy; ottavia.guglielmi@gmail.com (O.G.); sgarbarino.neuro@gmail.com (S.G.)

**Keywords:** sleep, sleep disorders, big data, OMICS sciences, connectomics, wearable sensors, behavioral informatics, infodemiology, infoveillance, personalized sleep medicine, precision sleep medicine

## Abstract

Sleep disorders have reached epidemic proportions worldwide, affecting the youth as well as the elderly, crossing the entire lifespan in both developed and developing countries. “Real-life” behavioral (sensor-based), molecular, digital, and epidemiological big data represent a source of an impressive wealth of information that can be exploited in order to advance the field of sleep research. It can be anticipated that big data will have a profound impact, potentially enabling the dissection of differences and oscillations in sleep dynamics and architecture at the individual level (“sleepOMICS”), thus paving the way for a targeted, “one-size-does-not-fit-all” management of sleep disorders (“precision sleep medicine”).

“*[There are] three fundamental questions: what is sleep, what are its mechanisms, and what are its functions*?”

Allan Hobson

## 1. The Impact of Sleep and Sleep Disorders on Human Health

Since the dawn of mankind, humans have always been fascinated with sleep [1]: the medieval Sephardic Jewish philosopher Moses ben Maimon, commonly known as Maimonides or Rambam (1135–1204), an authentic pioneer of the modern sleep hygiene, recommended sleep at least up to 8 h and speculated about the origin of dreams long before the birth of psychoanalysis. Centuries later, the father of chronobiology, French Jean-Jacques d’Ortous de Mairan (1678–1771) in the eighteenth century and subsequently Nathaniel Kleitman (1895–1999) and his student Bruce Richardson in the forties, anticipated the current theory of the biological clock, carrying out experiments on plants and humans, respectively [1].

However, despite such a huge and impressive body of research, the precise mechanisms that regulate sleep are yet to be unraveled. Sleep research is of crucial importance in that sleep is not a mere byproduct of human evolution but is fundamental in ensuring health and well-being. Sleep has generally been considered in negative terms, e.g., as a reduction of consciousness, a decrease of wakefulness, or an inactive or idling state in which normal daily activities are suspended. It is now emerging as a neurophysiological condition with its own dignity and its peculiar features. Sleep is “of the brain, by the brain, and for the brain”, as maintained by the American psychiatrist Allan Hobson, who was born in 1933 [2].

Sleep disorders are classically conceived as impairments of the sleep architecture and dynamics affecting the sleep-wake cycle, but sleep alterations affect the whole psycho-physical health and well-being, resulting in a range of diseases including cardiovascular morbidity and malignancies. The relationship between sleep and health is more complex and bidirectional than commonly thought [3]. Sleep has an influence on the endocrinological and immunological functions and systems, among others [4]. In order to better understand the relationship between sleep and health, a new approach (as will be delineated in the following paragraphs) is urgently required. This novel theoretical framework should encompass all aspects of human life, “from cradle to grave”, in that “children are not little adults” [5], and each stage has its peculiar aspects.

## 2. Epidemiology of Sleep Disorders: A Holistic View

The burden generated by sleep disorders is extremely relevant as they impose marked societal and economic costs in terms of direct/indirect healthcare and welfare costs [6,7,8]. Sleep disorders have reached epidemic proportions worldwide, affecting the youth as well as the elderly, crossing the entire lifespan in both developed and developing countries [9]. About 25% of all children experience some type of sleep problem at least once during childhood [10]. The “INDEPTH WHO-SAGE Study”, which recruited more than 40,000 older adults from eight countries across Africa and Asia, has shown a rather high prevalence of sleep problems among women and older age groups [11].

Profound changes in the current economy and labor market in an increasingly frantic society (“the post-modern 24 h society”), which have led to fragmentation of work, part-time jobs, night shifts, and the widespread use of the new media, new information, and communication technologies (ICTs), may have contributed to the increased rate of sleep disorders [12,13]. Light emitted from screens is believed to disrupt the users’ circadian rhythm even though a recent investigation has shown that the social cognition implied in the usage of social networking sites may play a major role [14].

Furthermore, cultural and social habits have influence on sleep architecture and dynamics, underlining the importance of considering the socio-cultural context [15,16,17,18]. Besides race and ethnicity, religiosity/spirituality is another emerging variable that has been found to impact sleep [19,20]. According to an initial review of the existing scholarly literature and conceptual model, religious involvement should be considered a determinant of sleep-related outcomes. Some studies seem to suggest that more religious adults report healthier sleep outcomes when compared to their less religious counterparts. Religiosity seems to help in coping with psychological distress, protecting from substance use, stress exposure, and allostatic load [20].

However, these aspects are generally poorly studied and overlooked by researchers. Further studies are needed to fill in the gaps of knowledge.

## 3. Big Data

Big data refer to extremely large data sets that, due to their complexity and high heterogeneity, cannot be analyzed with conventional techniques (e.g., multivariate regression analyses) but require particular computational efforts (“big data analytics”) in order to be effectively handled, manipulated, and integrated, which would allow for the extraction of significant patterns in terms of trends, interactions, and associations [21,22,23,24].

Big data are classically characterized by three V: velocity (referring both to the speed of data acquisition and data processing, big data as “fast data”), volume (relating to the amount of information), and variety (referring to the number of sources that can generate big data) [24].

There exist different kinds of big data, depending on their sources, including: (i) wet-lab techniques (and recent OMICS-based approaches like genomics, proteomics, metabolomics/metabonomics, methylomics, epigenomics, etc.); (ii) imaging (polysomnography); (iii) sensors (wearable sensors in particular, besides the classical wrist actigraphy); and (iv) digital and computational sources (internet, smart phones, and other mobile devices) [21].

According to Dolley, big data can be used for a variety of purposes such as performing disease surveillance and signal detection, predicting risk, targeting treatment interventions, and, last but not least, understanding disease [22].

The different types and sources of big data in the field of sleep research are briefly overviewed in Table 1 and explained in detail in the following paragraphs.

## 4. Entering Phase VI: A New Era for Sleep Medicine

The prominent sleep scientist William Charles Dement, who was born in 1928, proposed to subdivide sleep medicine into five phases, namely:

(i) the first phase (prehistoric), from inception up to 1952, an era characterized by non-scientific views and hypothesis about sleep;
(ii) the second phase, from 1952 to 1970, in which the scientific research or fascinating “journey through the night” began (discovery of rapid eye movement or REM sleep);
(iii) the third phase, from 1971 to 1980, devoted to the study of the determinants of sleepiness and day alertness, leading to the birth of the first scientific societies and scholarly organizations;
(iv) the fourth phase, from 1981 to 1990, in which new pharmacological and non-pharmacological treatments for sleep disorders emerged together with the awareness of the implications of sleep in terms of public health; and, finally,
(v) the fifth phase, from 1991 to 2000, in which the diagnosis of sleep disorders were brought to the mainstream society and the healthcare systems [1,25].

Sleep medicine is now entering phase VI, the era of personalized/individualized management and treatment [26], and the era of big data.

## 5. Sleep and Epidemiological Big Data

High quality longitudinal surveys involving repeated observations of the same variables/subjects over relatively long periods of time can capture variations in sleep architecture and dynamics from a neuro-developmental standpoint.

It should be noted that data derived from population- or communities-based studies can be integrated with data from molecular assays and/or neuro-imaging approaches.

Recently, for instance, the UK “Cambridge Centre for Ageing and Neuroscience” (Cam-CAN) cohort study in East Anglia/England collected self-reported health and lifestyle questions and objective measures from 2406 healthy adults, aged 18 to 98 years. Better self-reported sleep was found to be associated with better health outcomes, strongly associated for mental health and moderately for cognitive and physical health. Latent class analysis (LCA) identified four sleep types, namely “good sleepers” (68.1% of the sample, generally in middle age), “inefficient sleepers” (14.01%, most frequent in old age), “delayed sleepers” (9.28%, generally in adulthood) and “poor sleepers” (8.5%, most frequent in old age), even though there is little evidence for interactions between sleep quality and age on health outcomes [12,27].

However, sleep habits are not routinely explored. Studies could be integrated and merged, creating larger archives, in order to increase statistical power and obtain more robust and stronger scientific evidence.

## 6. Sleep and Molecular Big Data

The biological makeup of the individual has a deep impact on sleep habits. More in detail, the biological clock is located at the level of the suprachiasmatic nuclei of the anterior hypothalamus. This clock finely tunes and modulates the timing of sleep, wakefulness, and waking cognitive and neurobehavioral functioning based on a two-process model consisting of a sleep homeostatic process and a circadian process [28].

The gene-candidate approach and bioinformatics analyses enabled researchers to identify genetic loci and polymorphisms putatively associated with different features of sleep, including genes encoding for period circadian protein homolog 3 (PER3), human leukocyte antigen DQB1, adenosine deaminase (ADA), adenosine A2a receptor (ADORA2A), catechol-*O*-methyltransferase (COMT), dopamine transporter (DAT), dopamine receptor D2 (DRD2), tumor necrosis factor alpha (TNF-α), and basic helix-loophelix family member e41 (BHLHE41/DEC2), among others [29].

Another important polymorphism is given by sequence variations involving the gene encoding for the vitamin D receptor (VDR), which has been linked to the development of obstructive sleep apnea (OSA) [30].

In genomics and post-genomics studies, the development and implementation of adequate bioinformatics infrastructures and web-based resources is of fundamental importance. Recently, scholars created a tool devoted to accelerating research and facilitating the dissemination of information concerning heart, lung, blood, and sleep (HLBS) disorders—the HLBS population genomics (HLBS-PopOmics) knowledge base, which is an online, continuously updated, searchable database [31].

A recent genome-wide association study (GWAS) conducted among 91,105 UK Biobank participants led to the discovery of 14 loci putatively associated with sleep duration, accounting for 0.39% of its variation [32].

Nillson and coauthors performed a within-subject randomized, blinded trial with 16 healthy subjects in order to investigate the impact of one night of total sleep deprivation on the blood methylome compared with that in normal sleep. Utilizing bioinformatics techniques such as gene set enrichment analysis, authors identified the Notch and Wnt signaling pathways as being implied in the mechanisms at the basis of total acute sleep deprivation [33].

Sophisticated and advanced biostatistics, including machine learning techniques, can be applied to genomics data, leading to the successful identification of new biological circadian clock components, such as the gene CHRONO (computationally highlighted repressor of the network oscillator) [34].

## 7. Sleep and Signaling-Based Big Data

In the twenties, the German psychiatrist Hans Berger (1873–1941) invented electroencephalography (EEG) and discovered the alpha wave rhythm, also known as the “Berger wave” [35].

Currently, polysomnography offers a wealth of physiological information, informing clinical decision-making and clinical research. Large sleep-related datasets are increasingly available for public analysis. Some examples are the “National Sleep Research Resource” (NSRR), a new “National Heart, Lung, and Blood Institute” (NHLBI) resource designed ad hoc to provide access to big data to the sleep research community, PhysioNet (accessible at www.physionet.com), offering free web access to large collections of recorded physiologic signals, and the “Montreal Archive of Sleep Studies” (MASS) [36,37].

Yetton and collaborators exploited a Bayesian network-(BN) based approach by thoroughly investigating the influence of age, sex, body mass index (BMI), time of day, and sleep time on static (i.e., minutes in stage and sleep efficiency), and dynamic measures of sleep architecture (including transition probabilities and stage duration distributions). For this purpose, they used a large dataset of slightly more than 3200 nights from a non-clinical population. Multi-level regression analyses showed that sex impacted on the duration of all non-rapid eye movement (NREM) stages, and age had a curvilinear relationship with “wake after sleep onset” (WASO) and “slow-wave sleep” (SWS) minutes. Moreover, BN modeling revealed that sleep architecture depended on time of day, total sleep time, age, and sex, but was not influenced by BMI. Older adults, and males in particular had shorter bouts (more fragmentation) of Stage 2 (SWS), and they exhibited transition to these stages less frequently. Furthermore, authors showed that the next sleep stage and its duration could be optimally predicted by the knowledge of the prior two stages and age [38].

While wavelet analysis (WA) has been (and still is) an integral part in the understanding of EEG, new artificial intelligence-based approaches like artificial neural networks (ANNs) and support vector machine (SVM) are emerging as promising tools for providing an accurate and reliable interpretation of EEG.

ANNs are an example of computational models inspired by the human brain architecture. In fact, they can be imagined as a web formed of interconnected cells. These simulate the biological functions of neurons; they receive inputs, process them, and generate outputs, transmitting electric signals to each other. In ANNs, knowledge of the problem is therefore distributed among the neurons and connection weights of links between them. This parallel nature of the system contributes to a global behavior that is defined as emergent. The unique properties of ANNs explain their computational power. Although many types and topologies of ANNs exist, one of the most common patterns consists of a set of interconnected nodes (termed also as neurodes, processing elements, or units) grouped in multiple layers in which input nodes and output nodes have clinical correlates. Hidden nodes enable ANNs to approximate non-linear functions, but, differently from input and output nodes, they do not have any clinical correlate. The nodes are connected by links, and each link has an associated numeric weight that can be finely tuned based on experience or on any learning algorithm. This network is “trained”, for example, by exposure to inputs paired with known outputs. An exposure cycle is called “epoch” and is iterated until the best solution is found, and “learning” occurs when the inputs are strong enough to pass a threshold/cutoff in such a way that they activate the nodes and the weights between nodes are modified and adjusted according to feedback (supervised learning). Other types of learning are unsupervised learning and reinforcement learning, in which data are unlabeled or input/output pairs are not presented. Once the model is trained, it can then be tested against novel records to predict outputs until the desired output is finally obtained. ANNs, being advanced predictive modeling, are characterized by several advantages, including non-linearity, fault tolerance, universality, real-time operation, tunable parameters, and emergent, adaptive ability of learning. Therefore, ANNs are quite suitable algorithms for modeling complex non-linear relationships in health-care research. They offer a unique way to model complex systems in that they can incorporate variability and uncertainty and integrate a variety of data, both nominal and ordinal data [39].

SVM is a discriminative classifier formally defined by a separating hyperplane. In other words, given labeled training data (supervised learning), the algorithm outputs an optimal hyperplane, which enables the categorization of new examples.

ANNs and SVM can integrate and enhance WA, whereas WA-based state-of-art methods can be effectively utilized in feature generation and dimension reduction together with next-generation tools, such as the neighboring component analysis (NCA) and other techniques combining both linear and non-linear feature selection methods. ANNs and SVM can achieve accuracy up to 90.30% and 89.93%, respectively, in properly classifying EEG patterns and sleep scoring [40].

Concerning sleep disorders, Khandoker and collaborators utilized SVM to diagnose OSA syndrome (OSAS), a syndrome characterized by excessive daytime sleepiness and associated with poor quality of life and significant cardiovascular morbidity. Authors managed to reach accuracy up to 100% on a subset of recordings and up to 92.85% when replicating the findings on an independent dataset [41].

## 8. Sleep and Imaging-Based Big Data

Connectomics—combining diffusion tensor imaging (DTI) and resting-state functional magnetic resonance imaging (fMRI)—represents the frontier of neuro-imaging. It enables the capturing of the neural substrates underlying the physiology of sleep, as well as those correlating with clinical symptoms and the neurophysiologic mechanisms of various sleep disorders [42].

For instance, fMRI-based connectomics can be used to obtain an automatic staging of sleep, as shown by Tagliazucchi and coworkers. The approach relies upon multiclass classifiers properly combining with binary SVM classifiers that are able to discriminate between all pairs of sleep stages. It is based on linear correlation values between 20 cortical regions identified using independent component analysis (ICA) and two regions located at the level of the bilateral thalamus. This approach can reach accuracies over 80% using epochs as short as 60 s, thus properly modeling vigilance states [43].

Concerning sleep disorders, Wu et al. used connectomics to characterize a sample of 44 primary insomnia patients in terms of topologic alterations of the anatomical network architecture. Patients were age-, gender-, and education level-matched with 46 healthy controls. Authors found that primary insomnia patients exhibited a small-world architecture with lower global and local efficiencies when compared to controls. Patients demonstrated unique hub nodal properties in the right limbic cortico-basal-ganglia circuit, with up to five disrupted sub-networks observed at the level of the limbic cortico-basal-ganglia circuit and left default-mode networks, significantly correlating with disease duration and clinical characteristics [44].

Kaufmann and coworkers utilized connectomics to investigate the mechanisms underlying lack of sleep. They recruited 60 young adult male participants, 41 of which underwent total sleep deprivation. Authors found that sleep deprivation dramatically impacted the connectivity of various resting-state networks, such as dorsal attention, default mode, and hippocampal networks [45].

Cheng and collaborators utilized data from 1017 participants in the “Human Connectome Project” with the “Adult Self-Report of Depressive Problems” section of the “Achenbach Adult Self-Report for Ages 18–59”, a survey combining self-reported sleep quality questionnaires and resting-state fMRI. The “Depressive Problems” scores were found to be correlated with functional connectivities between areas such as the lateral orbito-frontal cortex, cingulate cortex, precuneus, angular gyrus, and temporal cortex [46].

## 9. Sleep and Digital and Computational Big Data

Sleep-related infodemiology (a port-manteau of “information” and “epidemiology”) and infoveillance (a combination of “information” and “surveillance”), a term coined by Gunther Eysenbach to indicate “the science of distribution and determinants of information in an electronic medium, specifically the internet, or in a population, with the ultimate aim to inform public health and public policy” [47], is still in its infancy.

As stated by Pavel, behavioral informatics approaches “have the potential to optimize interventions through monitoring, assessing, and modeling behavior in support of providing tailored and timely interventions” [48].

Novel data streams (NDS) include web search data and social media updates [49,50]. They have been exploited to investigate nocturnal leg cramps, restless leg syndrome, insomnia, and other sleep disorders [51]. Ji and Kang documented an increase in internet sleep disorder related search volumes throughout the years [52]. Interestingly, Ingram and colleagues found significant seasonal trends for both snoring and sleep apnea internet search engine queries, with peaks in the winter and early spring [53].

An interesting study raised concerns about the quality and reliability of sleep disorder related websites; according to Pusz and Brietzke, OSAS-related web pages contained biased, advertisement-type information [54]. Sleep specialists and institutions/health authorities and bodies devoted to sleep research and/or sleep medicine have the onus of educating patients and being more active in disseminating high quality online material.

## 10. Sleep and Wearable Sensors- and Self-Quantification Systems-Based Big Data

Big data can also be generated and produced by wearable sensors, devices, and self-quantification systems, and can be analyzed using artificial intelligence techniques, some of which have been extensively mentioned before [55,56].

Analogous with concepts such as the “quantified self” (a term coined by Gary Wolf and Kevin Kelly in 2007), and the “quantified traveler” [57,58,59], we hereby introduce the novel concept of “quantified sleep”/“quantified sleeper”, indicating the feasibility of tracking and monitoring real-time sleeping habits utilizing non-invasive tools.

Winnebeck and colleagues performed the first large scale analysis of human sleep dynamics in real life by making use of longitudinal wrist movement recordings of more than 16,000 sleep bouts from a sample of 573 subjects. Whereas no sex differences could be found, age and occupational features (such as shift work) were found to significantly impact sleep dynamics. The impacts were especially high on decline rates and ultradian amplitude, with no effect on ultradian period and phase [60].

In the effort to achieve an automatic sleep stage classification, which is of crucial importance for long-term sleep monitoring, Zhang and coworkers exploited wearable devices utilizing a two-phase approach—multi-level feature learning and recurrent ANNs-based classification. Authors were able to subdivide sleep into five stages (i.e., wake, non-rapid eye movement or NREM 1–3, and REM) with satisfactory precision and recall [61].

Concerning sleep disorders, Le et al. developed a Dirichlet process-based mixture Gaussian process (DPMG) model to predict the onset of OSA episodes based on the analysis of complex cardio-respiratory signals acquired by means of a custom-designed wireless wearable multisensory suite. Authors managed to achieve a good accuracy for predicting OSA [62]. Notably, this model can also be utilized to adjust the treatment of continuous positive airway pressure (CPAP) according to the specific features of the patient.

**Figure 1:**
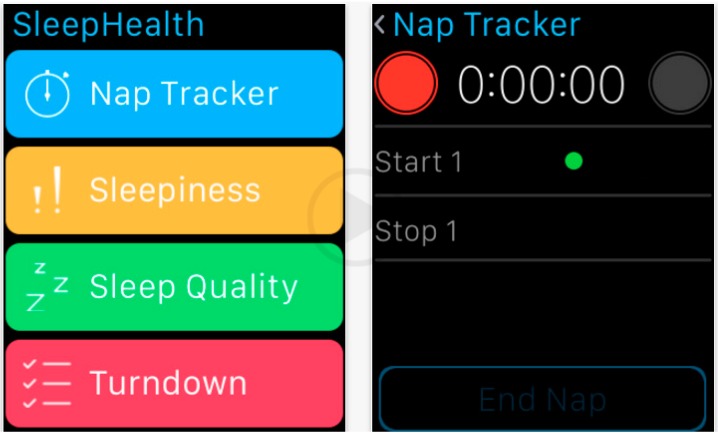
The “SleepHealth” application developed by the collaboration between the “American Sleep Apnea Association” (ASAA) and the “International Business Machines Corporation” (IBM).

Recently, the “American Sleep Apnea Association” (ASAA) and the “International Business Machines Corporation” (IBM) have decided to collaborate together and have launched the “SleepHealth Mobile Study”, the first patient-led sleep study based on Apple ResearchKit, which is an open-source software framework that facilitates the development of ad hoc applications (Figure 1).

However, interestingly, Baron and collaborators warned against the potential risk of orthosomnia—that is to say, the intentional behavior of utilizing sleep-tracking devices in the absence of sleep disorders in order to optimize daily cognitive functioning, being aware of the relationship between sleep and daytime fatigue [63].

## 11. An Executive, Practical Summary: Potential Roles of Big Data for the Sleep Physician

Specifically concerning the field of sleep medicine, big data can be used for performing sleep disorder surveillance, performing health surveillance in night-shift workers, signal detection/sleep disorder phenotyping (primary insomnia, OSA, post-influenza vaccination narcolepsy) [64,65], predicting risk (acute effects of sleep deprivation) [62,66], targeting treatment interventions (for example, in patients with OSA) [62,66,67], and, last but not least, understanding the neuro-physiological mechanisms underlying the sleep architecture and dynamics, as well as the basis of sleep disorders [22]. These are briefly summarized in Table 2.

## 12. Strengths, Pitfalls, and Future Prospects

Big data require adequate infrastructure with sufficient power to acquire, collect, store, and process data. Specifically, in the field of sleep research, some researchers have developed the “Comparative Outcomes Management with Electronic Data Technology” (COMET) platform, a scalable and extensible research infrastructure [68].

Recent achievements in the field of sleep medicine bioinformatics, including the adaptation of the “Minimal Domain of Discourse” (MiDas) algorithm to automatically extract sleep-related concepts, are expected to contribute to advancing sleep science [69].

Strengths include: (i) the data-driven approach, (ii) the feasibility of acquiring and monitoring sleeping habits in real-time, (iii) the possibility of studying sleep and sleep disorders at the individual level, and (iv) the predictive power of big data-based computational techniques. On the other hand, pitfalls derived from the usage of big data include (i) the extreme complexity and heterogeneity of certain datasets, and (ii) privacy and other ethical issues.

## 13. Big Data to Improve Sleep on a Population Level

Poor and/or inadequate sleep has important implications in terms of all-cause mortality, morbidity, health-related perceived quality of life, and occupational safety and well-being. In the USA, ensuring a proper amount of hours dedicated to sleep has been recognized as one of the priority targets of the “Healthy People 2020” plan [70]. Big data-based datasets, biobanks, and large scale epidemiological surveys can be exploited to inform policy and intervention at both the behavioral levels and the systems levels (for instance, regulating working schedules and rotating shifts in order to optimize sleep hygiene). Therefore, big data can be potentially used to mitigate the burden of patients suffering from sleep disorders, help people sleep better, and create change at a societal level.

## 14. Conclusions

Far from being a mere passive “idling state”, as wrongly believed in the past, sleep is a complex, widespread, multi-factorial, non-linear phenomenon that is influenced by an array of parameters, including biological parameters (in terms of genomics, epigenomics, and postgenomics signatures) as well as developmental, behavioral (lifestyles, cultural habits), environmental, and clinical variables.

Such a view requires the adoption of a new approach, one that is able to overcome the drawbacks and limitations that plague the classical mechanistic/reductionist frameworks in favor of a “systems sleep medicine” point of view.

“Real life” and real-time behavioral (sensor-based), molecular, digital/computational, and epidemiological big data represent the sources of an impressive wealth of information that can be exploited in order to advance the field of sleep research [70,71,72].

It can be anticipated that big data will have a deep impact, potentially enabling us to answer the “three fundamental questions: what is sleep, what are its mechanisms, and what are its functions?” [2]. Big data could help dissect the differences and oscillations of sleep dynamics at the individual level (“sleepOMICS”), paving the way for a targeted “one-size-does-not-fit-all” management of sleep disorders (“precision sleep medicine”).

## Figures and Tables

**Table 1:** An overview of the sources of big data in the field of sleep research.

Type of Big Data	Definition	Examples
Epidemiological big data	Health-related perceived quality of life data obtained by administration of questionnaires; these data can be integrated with/supplemented by molecular/imaging data	Longitudinal surveys
Molecular big data	Data acquired by means of wet-lab techniques (OMICS assays)	GWAS
Signaling-based big data	Real-time data acquired through biomedical devices	Clinical polysomnography
Imaging-based big data	Data acquired by means of neuro-imaging techniques	fMRI-based connectomics
Digital and computational big data	Real-time acquired data related to digital/computational habits (internet searches)	Novel data steams (Google Trends, Wikipedia, social networks, etc.)
Sensors-based big data	Real life, real-time acquired data from wearable sensors, enabling self-tracking and monitoring	Actigraphy, wearable sensors, self-quantification systems

Abbreviations: fMRI (functional magnetic resonance imaging); GWAS (genome-wide association studies).

**Table 2:** An overview of the sources of big data in the field of sleep research.

Potential Role of Big Data	Definition	Examples
Performing sleep disorder surveillance/performing health surveillance in night-shift workers and signal detection/sleep disorder phenotyping	Utilizing big data for diagnosing sleep disorders or for surveillance purposes	Primary insomnia OSA Post-influenza vaccination narcolepsy
Predicting risk	Utilizing big data in order to predict risk of developing sleep disorders or to predict impending episodes of sleep disorders	Acute effect of sleep deprivation OSA Impending OSA episodes
Targeting treatment intervention/predicting the effects/ending of a treatment	Using big data to personalize treatment and understand determinants of therapeutic success/failure	Predictors of CPAP therapy ending in the first year of treatment
Understanding neuro-physiological mechanisms of sleep and neuro-physiopathological basis of sleep disorders	Utilizing big data to explain and dissect the mechanisms of sleep	Automatic sleep staging Determinants of sleep architecture and dynamics

Abbreviations: OSA (obstructive sleep apnea), CPAP (continuous positive airway pressure).
